# Experimental Validation of an Immune Cell Infiltration Signature in Psoriasis: Translating Computational Modeling to In Vivo Efficacy

**DOI:** 10.1002/jcla.70203

**Published:** 2026-03-19

**Authors:** Tingjin Zheng, Rong Xu, Jianming Zhang, Xiujuan Wen, Hao Huang, Hongfeng Tang, Zhishan Zhang, Chong Zeng

**Affiliations:** ^1^ Department of Clinical Laboratory Quanzhou First Hospital Affiliated to Fujian Medical University Quanzhou City Fujian China; ^2^ Department of Pharmacy Quanzhou Medical College Quanzhou City Fujian China; ^3^ Medical Research Center & Dermatology The Eighth Affiliated Hospital, Southern Medical University (The First People’s Hospital of Shunde, Foshan) Foshan Guangdong China

**Keywords:** immune cells infiltration, immune microenvironment, inflammatory, psoriasis

## Abstract

**Objective:**

The psoriatic immune microenvironment (PIME) is central to psoriasis pathogenesis, yet its mechanistic drivers are incompletely defined. This study aimed to delineate immune cell infiltration patterns and identify pivotal disease‐related immune genes through a systematic analysis of the PIME.

**Methods:**

We evaluated the infiltration levels of 28 immune cell subtypes in 11 psoriasis‐related microarray datasets using single‐sample gene set enrichment analysis (ssGSEA). Subsequent differential expression, consensus clustering, and weighted gene co‐expression network analysis (WGCNA) were employed to identify key genes. These findings were validated using human psoriatic tissue samples and an imiquimod‐induced murine psoriasis model to construct a predictive model termed IMscore.

**Results:**

Our analysis identified five pivotal immune‐related differentially expressed genes (ImDEGs): CXCL8, CXCL9, CCL18, RGS1, and SAMSN1. A novel predictive model, IMscore, was constructed based on these ImDEGs to assess psoriasis risk. Furthermore, immune infiltration profiling and gene set enrichment analysis demonstrated that these ImDEGs are functionally associated with psoriasis‐related inflammatory pathways, validating the diagnostic utility of the IMscore framework.

**Conclusion:**

These results provide new insights into the immunological mechanisms underlying psoriasis and establish a multi‐gene signature with potential for improving early diagnosis and therapeutic development.

## Graphical abstract

We calculated the infiltration profiles of 28 immune cells within psoriatic immune microenvironment through single‐sample gene set enrichment analysis (ssGSEA) algorithm based on the eleven psoriasis related microarray datasets. Five immune‐related genes CXCL8, CXCL9, CCL18, RGS1, and SAMSN1 are associated with psoriasis, and establish a predictive model for psoriasis. Patient specimens and imiquimod induced psoriasis like mice model also confirmed the bioinformatics results.

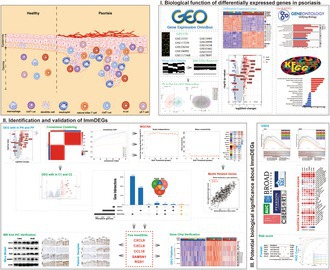

**Abbreviations**
AUC: area under the curve
BPs: biological processes
CCs: cellular components
CDF: cumulative distribution function
DEGs: differentially expressed genes
GEO: gene expression omnibus
GSEA: gene set enrichment analysis
ImDEGs: immune related different expression genes
IMQ: imiquimod
KEGG: kyoto encyclopedia of genes and genomes
MFs: molecular functions
MM: module membership
PAC: proportion of ambiguous clustering
PCA: principal component analysis
PIME: psoriatic immune microenvironment
PN: non‐lesional skin tissues
PP: lesional skin tissues GO: gene ontology
ROC: receiver operating characteristic
ssGSEA: single‐sample gene set enrichment analysis
TOM: topological overlap matrix
WGCNA: weighted gene co‐expression network analysis

## 1 Introduction

Psoriasis is a common chronic inflammatory skin disease characterized by keratinocyte hyperproliferation and immune cell infiltration in the dermis and epidermis, mediated by both innate and adaptive immune systems [1]. Affecting 2%–3% of the global population, it manifests as erythema, scaling, itching, and pain, with affected skin often covered by silvery scales [2, 3]. Beyond cutaneous symptoms, psoriasis confers a high risk of comorbidities, including cancer [4], metabolic syndrome [5], suicidality [6], and inflammatory diseases [7]. Although biologic therapies effectively control symptoms in many patients, challenges such as high treatment costs, adverse effects, and disease recurrence significantly compromise patient quality of life. Additionally, psoriasis is associated with severe psychiatric complications [6]. Elucidating the pathogenic mechanisms underlying psoriasis progression is therefore critical to identifying reliable biomarkers for precision therapy.

As a complex autoimmune disorder, psoriasis involves dynamic interactions between multiple immune cell subsets within the psoriatic immune microenvironment (PIME). Immune cells including mast cells, neutrophils, monocytes, macrophages, natural killer (NK) cells, T helper (Th)1, Th2, Th9, Th17, Th22, T follicular helper (Tfh), regulatory T (Treg), and B cells have all been implicated in PIME infiltration [8, 9, 10]. These cells synergistically secrete pro‐inflammatory cytokines (IFN‐γ, TNF‐α, IL‐17A, IL‐22, and IL‐23), which drive and sustain psoriatic inflammation. These cells work synergistically to release cytokines, such as IFN‐γ, TNF‐α, IL‐17A, IL‐22, and IL‐23 which induce and propagate psoriatic inflammation [11]. Consistent with this, biologic agents targeting pro‐inflammatory cytokines or lymphocyte activation have shown remarkable clinical efficacy in psoriasis management [12]. Given their central role in PIME, immune cells—particularly those infiltrating the psoriatic microenvironment—warrant prioritization in preclinical model development for identifying optimal prognostic biomarkers.

With advancements in genomic technologies, bioinformatics analysis of gene expression profiles has emerged as a cornerstone of molecular mechanistic studies, playing an increasingly pivotal role in discovering disease‐specific biomarkers. Among these tools, weighted gene co‐expression network analysis (WGCNA) stands out as a powerful systems biology approach. Unlike traditional differential gene screening methods, WGCNA overcomes their limitations by enabling prediction of co‐expressed gene functions and identification of hub genes central to disease pathogenesis. Notably, WGCNA has been successfully applied to diverse diseases, including non‐alcoholic fatty liver disease [13], prostate cancer [14], ischemic stroke [15], dilated cardiomyopathy with heart failure [16], and rheumatoid arthritis [17]. This versatility underscores its utility in elucidating gene regulatory networks and pinpointing key drivers of disease progression.

Against this backdrop, the present study leverages WGCNA alongside other algorithms (GSEA) to characterize immune infiltration patterns within the PIME. By integrating multi‐omics data, we aim to develop a risk stratification signature for psoriasis, which may optimize precision therapy and improve clinical outcomes.

## 2 Materials and Methods

### 2.1 Recruitment of Participants and Sample Collection

A total of 22 skin biopsy samples was included: 10 samples from control patients and 12 samples from psoriasis patients who were firstly diagnosed with psoriasis vulgaris by pathologic examination (Table 1), and without rheumatoid arthritis, alopecia areata, diabetes, etc. All these samples were used for subsequent immunohistochemical validation experiments and were not subjected to transcriptomic profiling. This study was approved by the Ethics Committee of Shunde Hospital, Southern Medical University (The First People's Hospital of Shunde) (no. KYLS20231055) in accordance with the declaration of Helsinki principles. Informed consent was obtained for all procedures.

**TABLE 1:** Baseline demographics of psoriasis patients and healthy individuals.

Sample ID	Sample type	Gender	Age	PASI score
P2349133	Psoriatic lesional skin	Male	35	1.3
P2349139	Psoriatic lesional skin	Male	25	1.4
P2348777	Psoriatic lesional skin	Female	33	1.5
P2348493	Psoriatic lesional skin	Male	27	1.8
P2347107	Psoriatic lesional skin	Female	34	1.6
P2340949	Psoriatic lesional skin	Male	36	1.7
P2337369	Psoriatic lesional skin	Male	37	0.9
P2337563	Psoriatic lesional skin	Female	28	1.4
P2330541	Psoriatic lesional skin	Male	35	1.8
P2333061	Psoriatic lesional skin	Female	42	1.2
P2319562	Psoriatic lesional skin	Female	32	2.2
P2318160	Psoriatic lesional skin	Male	36	1.3
P10117608	Psoriatic uninvolved skin	Male	44	0
P10117486_	Psoriatic uninvolved skin	Male	55	0
P10112671	Psoriatic uninvolved skin	Female	52	0
P772221	Psoriatic uninvolved skin	Female	40	0
P688444	Psoriatic uninvolved skin	Male	45	0
P10116207	Psoriatic uninvolved skin	Female	39	0
P10118640	Psoriatic uninvolved skin	Male	41	0
P828940	Psoriatic uninvolved skin	Female	45	0
P10119491	Psoriatic uninvolved skin	Male	35	0
P10119634	Psoriatic uninvolved skin	Female	28	0

### 2.2 Publicly Available Data Collection and Initial Preparation

The *getGEO* function in the *GEOquery* package [18] was used to obtain publicly accessible psoriasis‐related datasets from the Gene Expression Omnibus (GEO) database (https://www.ncbi.nlm.nih.gov/geo/), a widely recognized archive for gene expression data [19]. A total of 11 datasets derived from the Affymetrix GPL570 platform (Human Genome U133 Plus 2.0 Array) were obtained, namely, GSE106992 [20], GSE117239 [21], GSE117468 [22], GSE13355 [23], GSE14905 [24], GSE226244 [25], GSE30999 [26], GSE34248 [27], GSE41662 [27], GSE53552 [28], and GSE78097 [29]. Probe sets were reannotated by mapping all probes to the human genome (hg38) using SeqMaq [30]. Subsequently, batch effects were eliminated, and all datasets were merged into a cohesive GEO cohort. Distribution box plots and principal component analysis (PCA) were used to compare the before‐ and after‐effects of batch correction (Figure S1). The detailed baseline information of the 11 included datasets is summarized in Table S1.

### 2.3 Identification of Differential Expression Genes

The *limma* package was used to identify differential expressed genes (DEGs) between non‐lesional (uninvolved) skin tissues (termed PN) and lesional skin tissues (termed PP) from patients with psoriasis in the cohesive GEO cohort (|log_2_FoldChange| > log_2_ (3); adjusted *p*‐value < 0.05) [31]. The details of those DEGs are displayed in Table S2.

### 2.4 Functional Analyses

Gene Ontology (GO) is a common approach to functional enrichment analysis of genes in large‐scale studies [32]. It encompasses three distinct categories as follows: cellular components (CCs), molecular functions (MFs), and biological processes (BPs). In this study, the *clusterProfiler* package was used to perform GO analysis on DEGs [33], with *p*‐values of < 0.05 and *q*‐values of < 0.05 indicating statistically significant results. All details related to GO analysis are summarized in Table S3.

Gene set enrichment analysis (GSEA) [34] is used to assess the distribution patterns of genes within a pre‐defined set by analyzing a list of genes ranked based on their association with a particular phenotype. This method helps determine the contribution of the gene set to the phenotype. In this study, the *clusterProfiler* package was used to perform GSEA on DEGs [33], with the parameters being set as follows: seed, 2020; number of permutations, 1000; minimum number of genes per gene set, 10; and maximum number of genes per gene set, 500. The Benjamini–Hochberg method was used to adjust *p*‐values. The Molecular Signatures Database was used to obtain the “c2.all.v2023.1.Hs.entrez.gmt” gene set for GSEA. FDR values (*q*‐values) of < 0.25 and *p*‐values of < 0.05 indicated significant enrichment. All details related to GSEA are presented in Table S4.

### 2.5 Estimate the Infiltration State of Immune Cells

Single‐sample gene set enrichment analysis (ssGSEA) is a novel method for quantifying immune cell subsets based on gene expression data from various tissue types [35]. It has less noise and unknown mixture content than other methods, and the cell types are closely related. In this study, the R package *GSVA* was used to implement ssGSEA to evaluate the relative infiltration levels of 28 immune cell types in the PIME [36]. Differences in the infiltration levels of all immune cells were analyzed in the GEO cohort, and the results were visualized. All details related to immune cell infiltration analysis are presented in Table S5.

### 2.6 Consensus Cluster Method and Identification of Immune Subtypes

Based on their infiltration profiles, the 28 immune cell types were subjected to consensus clustering (a resampling‐based method) in the GEO cohort using the *ConsensusClusterPlus* package [37]. Subsequently, the consensus score matrix, cumulative distribution function (CDF) curve, proportion of ambiguous clustering (PAC) score, and Nbclust were used to determine the optimal number of clusters. The R package *limma* was used to identify differentially expressed genes between psoriatic subtypes in the GEO cohort, with the screening criteria set as |log_2_FC| values of > log_2_ (1.8) and *p*‐values of < 0.05. A total of 35 upregulated and 1 downregulated mRNAs were identified between the C1 and C2 subtypes in patients with psoriasis. The detailed results are shown in Table S6.

### 2.7 Weighted Gene Co‐Expression Correlation Network Analysis

Weighted gene co‐expression correlation network analysis (WGCNA) was performed to identify modules of highly correlated genes and analyze the inter‐relationships between the modules and their association with external sample traits [38]. An appropriate soft threshold power (*β*) was selected to develop a scale‐free network. Furthermore, the weighted adjacency matrix was converted to a topological overlap matrix (TOM), and the corresponding dissimilarity was assessed (1‐TOM). The dynamic tree cutting approach was used to identify modules. Specifically, lncRNA modules that showed the strongest correlation with immune clusters were selected for further analysis. Protein‐coding genes with both high gene significance (GS) and module membership (MM) values were defined as immune‐related genes. All details related to the immune‐related genes are presented in Table S7.

### 2.8 ImDEGs Based Diagnostic Model Construction

To construct a diagnostic model for psoriasis, DEGs1 (DEGs between PN and PP samples), DEGs2 (DEGs between the C1 and C2 subtypes in PP samples), and the immune‐related genes identified via WGCNA were intersected and the overlapping genes were selected as candidates for modeling (ImDEGs). Subsequently, logistic regression analysis was used to construct a risk score model based on the following formula [39]:

$$\text{IMScore}=\sum_{i}\text{Coefficient}\;\left(\mathrm{hub}\;{\text{gene}}_{i}\right)\times \text{mRNA Expression}\;\left(\mathrm{hub}\;{\text{gene}}_{i}\right)$$

In the above mentioned equation, coefficient (hub gene_*i*_) and mRNA expression (hub gene_*i*_) represent the survival correlation regression coefficient and expression value of each ImDEG, respectively. The coefficients of the 5 ImDEGs used to construct the risk score model are shown in Table S8, elucidating the contribution of each gene to the cumulative risk score.

### 2.9 Receiver Operating Characteristic (ROC) Curves

Receiver operating characteristic (ROC) curves serve as an efficient tool for assessing the diagnostic performance of a biomarker or gene set, with the area under the curve (AUC) values representing a quantitative measure of the predictive accuracy. Typically, an AUC value closer to 1 indicates superior diagnostic performance. In this study, the *multiple ROC* package was used to plot ROC curves to evaluate the diagnostic performance of the 5 ImDEGs and IMScore in the GEO cohort [40]. AUC values were computed to evaluate the respective predictive accuracies.

### 2.10 Imiquimod Induced Psoriasis‐Like Mouse Model and Experimental Design

Male C57BL/6J mice (6–8 weeks old) were purchased from Guangdong Medical Laboratory Animal Center (Guangdong, China). All mice were housed under specific pathogen‐free conditions and maintained on a 12‐h/12‐h light/dark cycle (lights on at 6:00 am and lights off at 6:00 pm) at a temperature of 21°C ± 2°C and humidity level of 60% ± 10%, with unrestricted access to food and water. All animal experiments were approved by the Animal Care and Use Committee of Shunde Hospital. All surgical procedures were conducted under anesthesia, and all efforts were made to minimize suffering.

Mice were randomly divided into two groups (*n* = 6 each group) and treated with a daily topical dose of 62.5 mg of imiquimod (IMQ) cream (5%) (Sichuan MedShine Pharmaceutical Co., H20030128) on the shaved back for 7 consecutive days [41]. Control mice were treated with the same dose of vehicle cream. Each mouse weight was weighed and recorded every day with an electronic balance. On day 8, the weight of each spleen was also measured. Skin tissues were taken from the sacrificed mice for extraction of protein and histological analysis.

### 2.11 Western Blot Assay

Mice skin tissues were lysed in the lysis buffer (RIPA, Beyotime, China) with PMSF as previously described [42, 43]. The total protein concentration was determined with BCA Protein Assay Kit (Beyotime, China). All samples were diluted in protein loading buffer, and heated to 95°C for 5 min. Equal amounts of protein were loaded in 12% sodium dodecyl sulfate‐polyacrylamide gel (SDS‐PAGE) and were then transferred from the gel to polyvinylidene fluoride (PVDF) membranes (Millipore, USA). After blocking with 5% BSA for 1 h at room temperature, the PVDF membranes were incubated with the indicated primary anti‐RGS1 (1: 1000), anti‐SAMSN1 (1: 1000), anti‐CXCL8 (1: 1000), anti‐CXCL9 (1: 1000) antibody antibodies overnight at 4°C. The PVDF membranes were incubated with HRP‐conjugated secondary antibodies followed by three washes with TBST. Proteins were visualized with enhanced chemiluminescence (Beyotime, China). GAPDH was act as loading control for each protein of target. Data were analyzed with Quantity One analysis software (Bio‐Rad).

### 2.12 Histological Analysis and Immunohistochemistry

The histological analysis was performed as previously described [44]. Briefly, human and mouse skin tissues were acquired and fixed in 10% paraformaldehyde for 48 h then embedded in paraffin. Skin tissues were cut into 5 μm thick sections and were stained with hematoxylin and eosin (H&E). H&E staining skin sections were captured using a Leica microscope (DM4B, Germany). For immunohistochemistry (IHC), the paraffin sections of skin tissue were dewaxed, hydrated, repaired with citric acid buffer, and incubated in 3% hydrogen peroxide solution for 15 min at room temperature to block endogenous peroxidase activity. Subsequently, sections were incubated with primary anti‐RGS1 (1: 100), anti‐SAMSN1 (1: 100), anti‐CXCL8 (1: 100), and anti‐CXCL9 (1: 100) antibodies in a humid chamber at 4°C overnight. After washing with phosphate buffer solution containing 0.1% Tween‐20 (PBST), the sections were incubated with the secondary antibodies (HRP‐goat anti‐rabbit IgG) at 37°C for 30 min, followed by diaminobenzidine color development for 5 min. Each section was observed using a Leica microscope and analyzed with image pro plus 6.0 (IPP 6.0; Media Cybernetics).

### 2.13 Data and Statistical Analysis

R (version 4.3.0, https://www.r‐project.org) and GraphPad Prism (version 8.3.0, Chicago, IL, USA) were applied for statistical analysis. For all analyses involving multiple hypothesis testing across genomic/transcriptomic datasets, *p*‐values were adjusted using the Benjamini‐Hochberg method to control the false discovery rate (FDR). Specifically, FDR correction was applied to genome‐wide differential expression analysis and gene set enrichment analysis. An adjusted *p*‐value (FDR) < 0.05 was considered significant unless otherwise specified. For targeted comparisons between patient subgroups, nominal *p*‐values are reported with the threshold noted. Each group size was *n* = 6 in animal experiments. GraphPad Prism 8 software was used to carry out statistical analysis. All the data are presented as mean ± SEM. Unpaired Student's *t*‐test was used for statistical analysis. *p* < 0.05 was considered statistically significant.

## 3 Results

### 3.1 Identification of DEGs and Functional Enrichment Analysis Associated With Psoriasis

**FIGURE 1:**
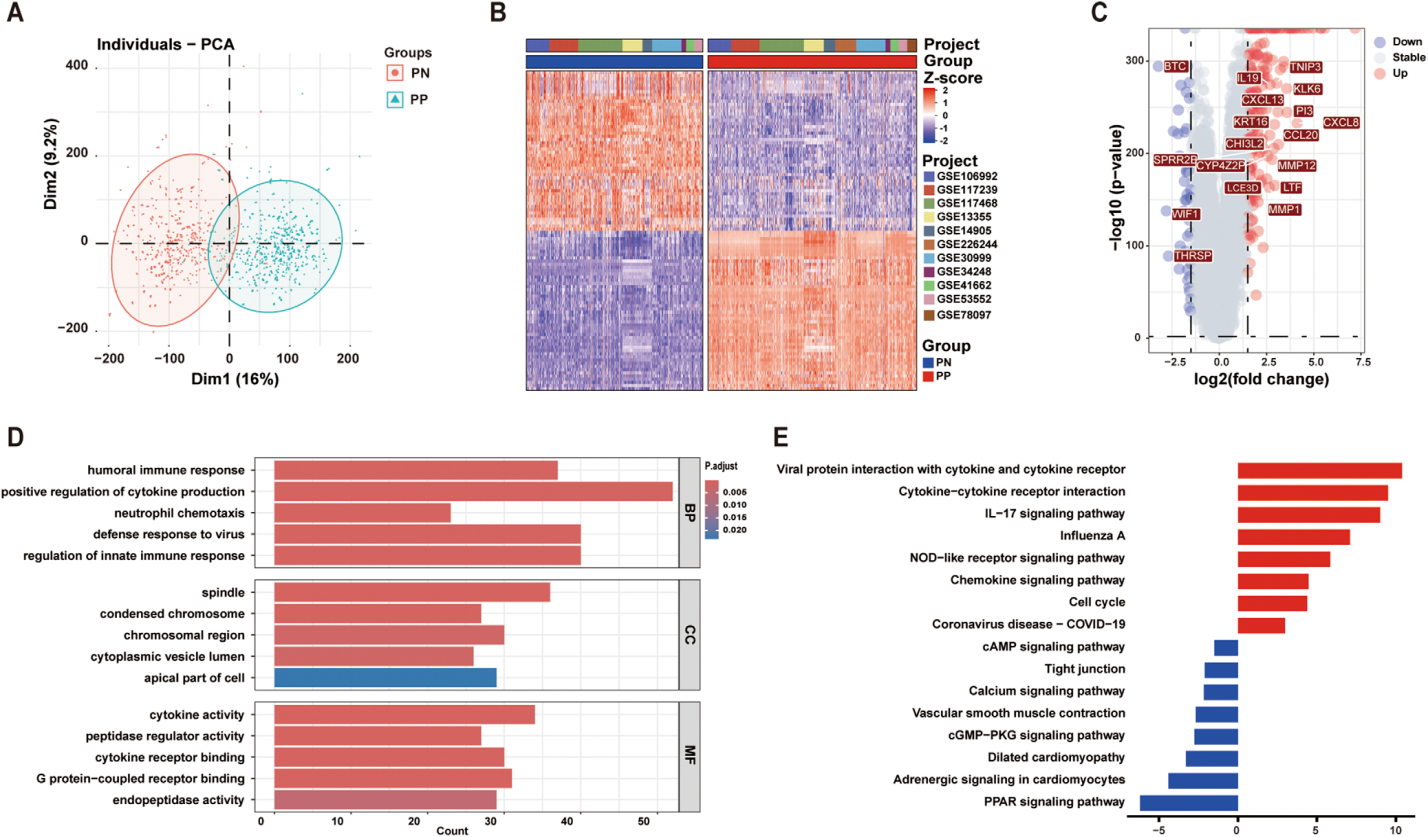
Identification of DEGs and functional enrichment analysis associated with psoriasis. (A) Principal component analysis (PCA) of psoriasis and control groups. (B) The heat map of DEGs in psoriasis and control groups. (C) The volcano map of DEGs in psoriasis and control groups. (D) GO enrichment analysis of DEGs. (E) KEGG pathway analysis of DEGs. PN, Non‐lesional skin tissues; PP, Lesional skin tissues; DEG, Differential expressed genes.

In this study, a total of 11 psoriasis‐related microarray datasets, GSE106992, GSE117239, GSE117468, GSE13355, GSE14905, GSE226244, GSE30999, GSE34248, GSE41662, GSE53552, and GSE78097, were obtained from the GEO database. These datasets collectively comprised 605 PP samples and 611 PN samples from patients with psoriasis. After quality‐control assessment, gene expression profiles were consistent across the 11 datasets (Figure S1), suggesting that any potential batch effects were successfully removed. PCA and hierarchical clustering revealed a clear distinction between PP and PN samples in the combined GEO cohort (Figure 1A).

A total of 208 DEGs were identified between PP and PN samples in the GEO cohort (DEGs1) (|log_2_FC| > log_2_ (3) and *p* < 0.05). A heatmap demonstrating the most significant DEGs, among them 176 genes were upregulated, and 32 genes were downregulated in the PP group. The DEGs were subjected to GO and kyoto encyclopedia of genes and genomes (KEGG) functional enrichment analyses to investigate their biological functions and signaling pathways (Figure 1B,C). The DEGs were significantly enriched in BPs associated with immune function, such as the humoral immune response, positive regulation of cytokine production, and regulation of the innate immune response (Figure 1D). KEGG analysis showed that the DEGs in the PP group were enriched in various pathways, including NOD‐like receptor signaling pathway, IL‐17 signaling pathway, and chemokine signaling pathway (Figure 1E). These findings suggested the presence of a highly inflammatory immune microenvironment in PP samples.

### 3.2 Identification of Psoriatic Subtypes Derived From Immune Infiltration Patterns

**FIGURE 2:**
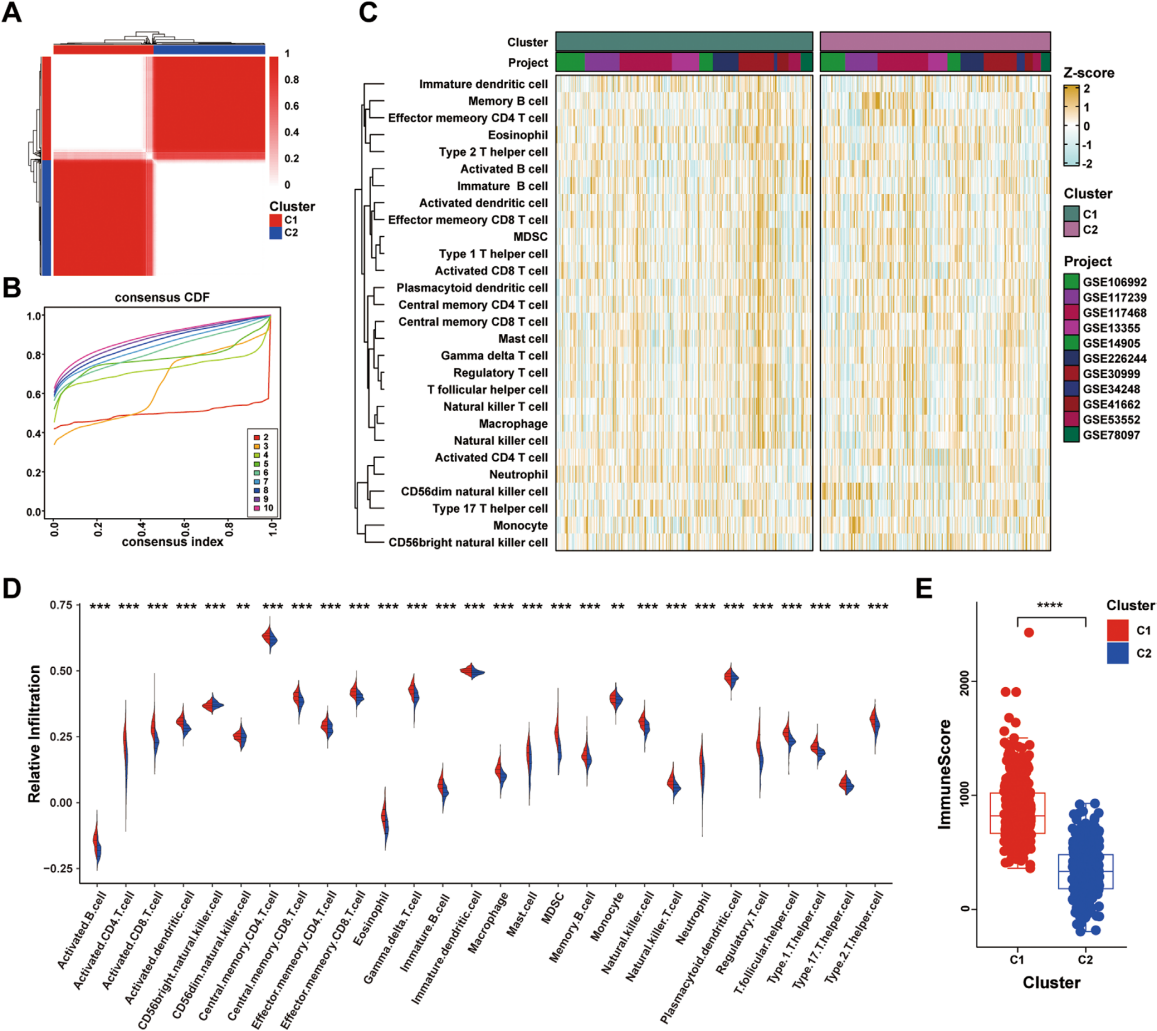
Identification of psoriatic subtypes derived from immune infiltration patterns. (A) The consensus score matrix of all samples when *k* = 2. A higher consensus score between two samples indicates they are more likely to be grouped into the same cluster in different iterations. (B) The CDF curves of consensus matrix for each *k* (indicated by colours). (C) The infiltration abundance of 28 immune cell subsets evaluated by ssGSEA for two clusters. (D) The distribution of 28 immune cell subsets infiltration between two clusters. (E) The distribution of immune score inferred by ESTIMATE algorithm between two clusters in the GEO cohort. Statistic test: Two‐sided unpaired t test. In boxplot graphs centre line indicates median, bounds of box indicate 25th and 75th percentiles, and whiskers indicate minimum and maximum. ****p < 0.0001. CDF, Cumulative distribution function.

To investigate the mechanisms underlying the development of psoriasis, the ssGSEA algorithm was used to evaluate the infiltration levels of 28 immune cell types in the GEO cohort. Based on the infiltration profiles of the immune cells, consensus clustering was performed, in which 605 PP samples were initially divided into *k* (*k* = 2–9) clusters. The CDF curves derived from the consensus score matrix and the PAC statistic indicated that the optimal number of clusters was 2 (Figure 2A,B). Consequently, PP samples were categorized into two psoriatic subtypes (C1 and C2) that demonstrated significant heterogeneity in immune infiltration levels. In particular, the C1 subtype had a markedly higher overall infiltration level than the C2 subtype (Figure 2C,D). Additional data from the ssGSEA algorithm demonstrated notable dissimilarities between the two subtypes based on the immune infiltration score (Figure 2E).

### 3.3 Construction and Module Analysis of the Weighted Co‐Expression Network

**FIGURE 3:**
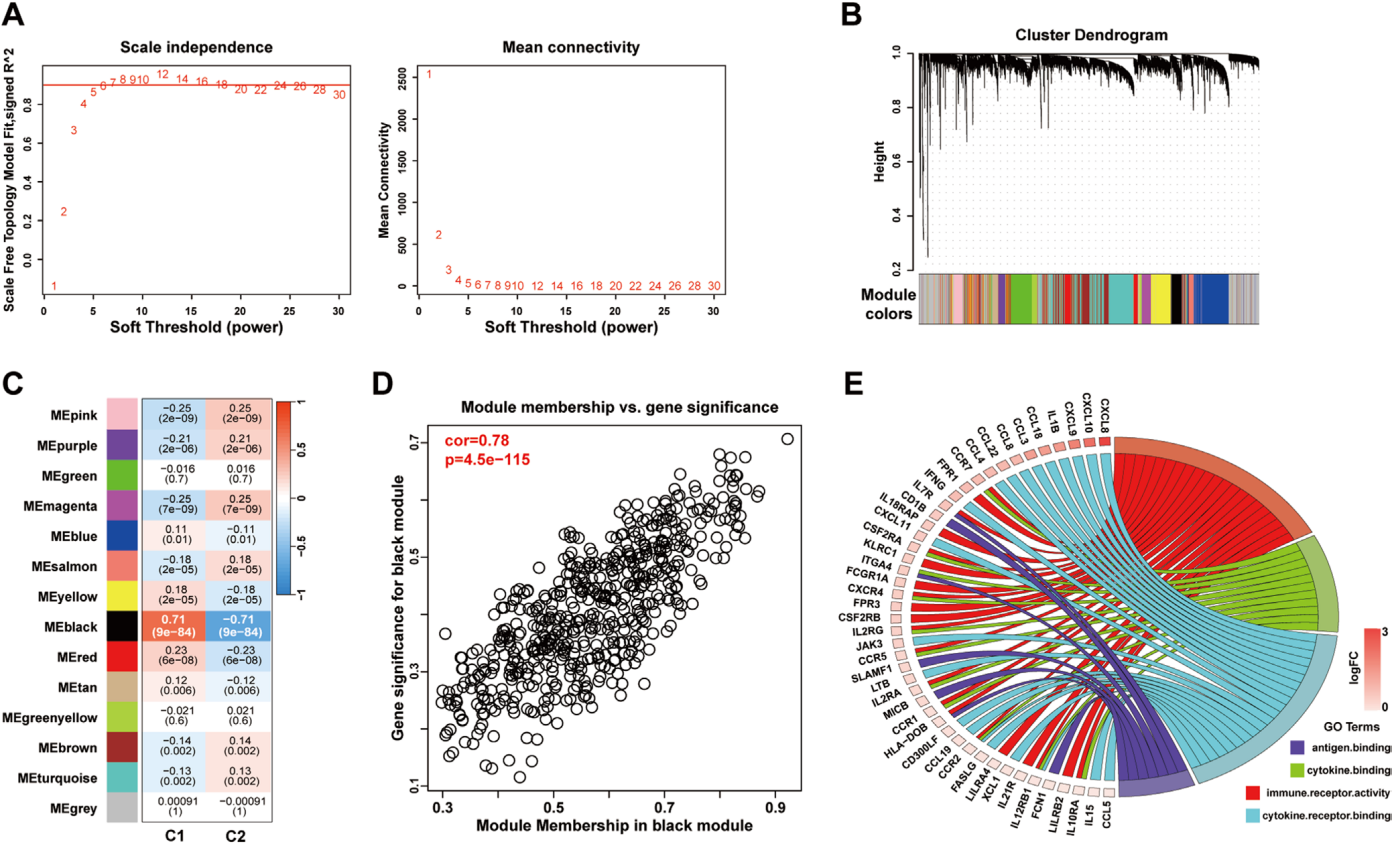
Construction and module analysis of the weighted co‐expression network. (A) Network topology analysis under various soft threshold power settings. Left: The X‐axis represents the soft threshold power, and the Y‐axis represents the fitting index of scale‐free topological model. Right: The X‐axis represents the soft threshold power, and the Y‐axis reflects the average connectivity (degree). (B) Clustering tree of genes with different similarities based on topological overlap, and assigned module colors. (C) Correlation analysis between module eigengenes and clinical traits. Each row corresponds to a module, and each column corresponds to a feature. Each cell contains the corresponding correlation and P values. This table is color‐coded according to the correlation of the color legends. (D) The high correlation between GS and MM in the black module (*p* = 0). Statistic test: Pearson's correlation coefficient, two‐sided unpaired t test. (E) Main GO terms enriched by the aforementioned genes within black modules. GS, Gene significance; MM, Module membership.

To precisely identify key immune‐related genes associated with psoriasis, WGCNA was performed on the C1 and C2 subtypes. A suitable power value (*n* = 5) for constructing a gene co‐expression network was obtained after the soft threshold *β* was set to 0.85 (scale‐free *R* ^2^ = 0.862) (Figure 3A). A total of 14 modules were generated, which are indicated by different colors (Figure 3B). The eigengene (the first principal component of gene expression within a module) was considered the representative of the module. Furthermore, the correlation between the modules and the two psoriatic subtypes (C1 and C2) was analyzed. The “black” module was found to have the strongest correlation with the psoriatic subtypes, with a correlation coefficient of 0.71 and a *p*‐value of 9e‐84 (Figure 3C). In the “black” module, the correlation coefficient between GS and MM reached 0.78, with a *p*‐value of 1.5e‐115, which suggested that the quality of the mRNA module was superior (Figure 3D). GO analysis validated the correlation between genes in the “black” module and immune‐related BPs and signaling pathways (Figure 3E). Therefore, the “black” module was identified as a key module associated with psoriasis. In addition, based on the immune infiltration patterns in the “black” module, 347 genes with GS values of > 0.3 and MM values of > 0.4 in the module were identified as hub immune‐related genes (Table S7).

### 3.4 Efficiency of Identified Hub Immune‐Related DEGs

**FIGURE 4:**
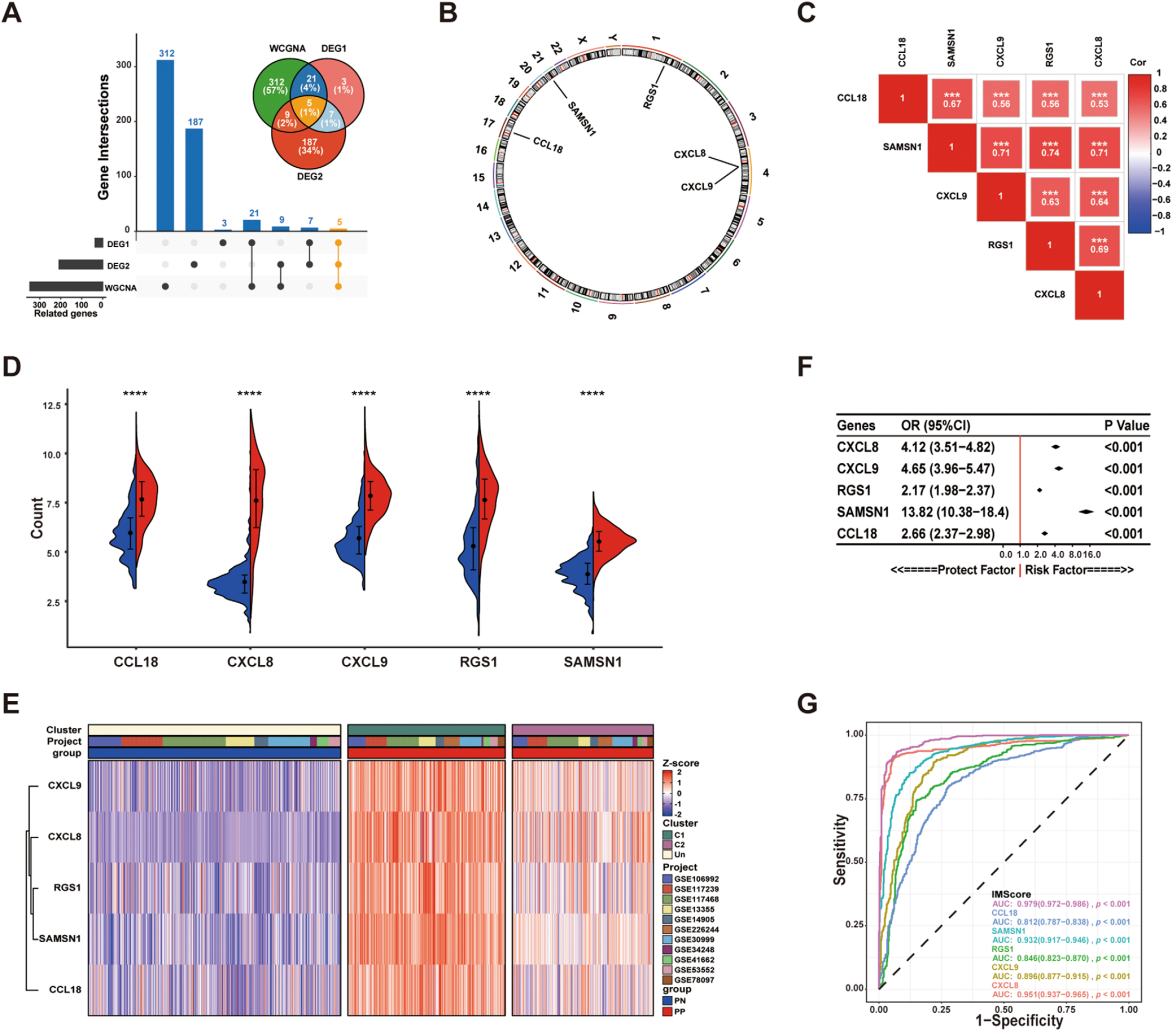
Efficiency of identified hub ImmDEGs. (A) Upset plots showing the number of identified genes from each method and their intersection. (B) Chromosome location map of ImmDEGs. (C) The expression level of ImmDEGs between psoriasis and control groups. (D) Heatmaps showing ImmDEGs expression in GEO cohort. (E) Correlation heat map of ImmDEGs; Red represents positive, Blue represents negative. The depth of the color indicates the strength of the correlation. (F) Forest plot showing the logistic regression analysis of ImmDEGs. (G) ROC curves of ImmDEGs and IMScore.

A total of 36 DEGs were identified between the C1 and C2 subtypes (DEGs2) (Table S6). These DEGs were intersected with the DEGs identified between PP and PN samples (DEGs1) and the 347 immune‐related genes identified via WCGNA, resulting in the identification of 5 hub immune‐related DEGs (ImDEGs) (Figure 4A). Circle diagrams were generated to visualize the locations of ImDEGs on human chromosomes. Both CXCL9 and CXCL8 were located on chromosome 4, whereas RGS1, CCL18, and SAMSN1 were located on chromosomes 1, 17, and 21, respectively (Figure 4B). Subsequently, the expression of each ImDEG was compared and visualized on a half‐violin plot and heatmap. The expression levels of all ImDEGs were higher in PP samples than in PN samples and in the C1 subtype than in the C2 subtype, suggesting the potential role of the genes in psoriasis (Figure 4C,D). The correlations between the ImDEGs were mostly positive, with SAMSN1 and RGS1 exhibiting the strongest positive correlation (Cor = 0.74) (Figure 4E).

To examine the correlation between the expression of ImDEGs (CXCL9, CXCL8, CCL18, SAMSN1, and RGS1) and the incidence of psoriasis, univariate and multivariate logistic regression analyses were performed in the GEO cohort. The results showed that the 5 ImDEGs might serve as risk factors for psoriasis (Figure 4F). Based on the co‐effects of ImDEGs, the risk score, termed ImScore, was calculated for all samples in the GEO cohort. Subsequently, ROC curves were plotted to evaluate the diagnostic efficacy of ImDEGs and ImScore. The AUC value of ImScore reached 0.979 (95% CI, 0.972–0.986), whereas that of the 5 ImDEGs reached 0.896 (95% CI, 0.877–0.915), 0.951 (95% CI, 0.937–0.965), 0.812 (95% CI, 0.787–0.838), 0.932 (95% CI, 0.917–0.946), and 0.846 (95% CI, 0.823–0.870), respectively (Figure 4G).

### 3.5 The Immune‐Related Pathways Involved in ImDEGs

**FIGURE 5:**
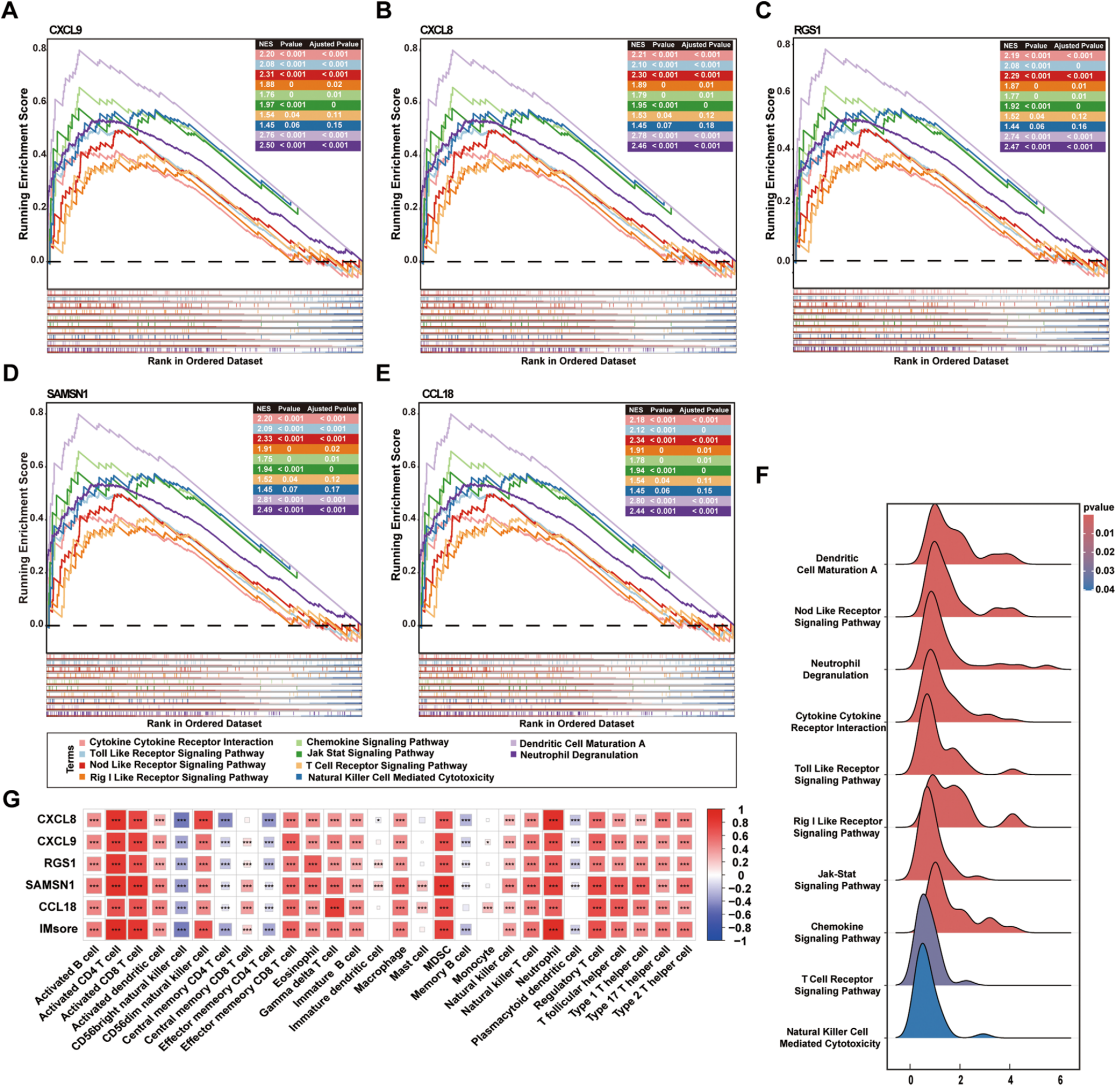
The immune‐related pathways involved in ImDEGs. (A‐E) GSEA plot showing five representative immune‐related pathways enriched for (A) CXCL9, (B) CXCL8, (C) RGS1, (D) SAMSN1, and (E) CCL18 in psoriasis. (F) Ridge plot showing the top 10 representative immune‐related pathways related to IMScore. (G) Heatmap showing the correlations between ImDEGs expression and immune cell subsets' infiltration.

To further elucidate the functions of the 5 ImDEGs, Pearson correlation analysis was performed between ImDEGs and all other genes. Genes positively associated with CXCL9, CXCL8, CCL18, SAMSN1, or RGS1 (*r* > 0.3, *p* < 0.05) were selected for subsequent enrichment analysis based on the C2.cp.v7.4.symbols.gmt reference gene set (Table S8). The results showed that all ImDEGs were significantly enriched in the chemokine signaling pathway, Rig‐I‐like receptor signaling pathway, Nod‐like receptor signaling pathway, Toll‐like receptor signaling pathway, T‐cell receptor signaling pathway, Jak–Stat signaling pathway, cytokine–cytokine receptor interaction, neutrophil degranulation, dendritic cell maturation, and natural killer cell‐mediated cytotoxicity (Figure 5A–E). Furthermore, ImScore was subjected to GSEA. As shown in the ridge plot in Figure 5F, ImScore was significantly enriched in the aforementioned 10 immune‐related pathways.

Furthermore, the expression of ImDEGs was positively correlated with the infiltration levels of several immune cell types, including neutrophils, MDSCs, natural killer cells, B cells, and T cell subpopulations, but negatively correlated with those of memory T cells and memory B cells (Figure 5G).

### 3.6 Expression Validation of ImDEGs With Clinical and Mice Samples

**FIGURE 6:**
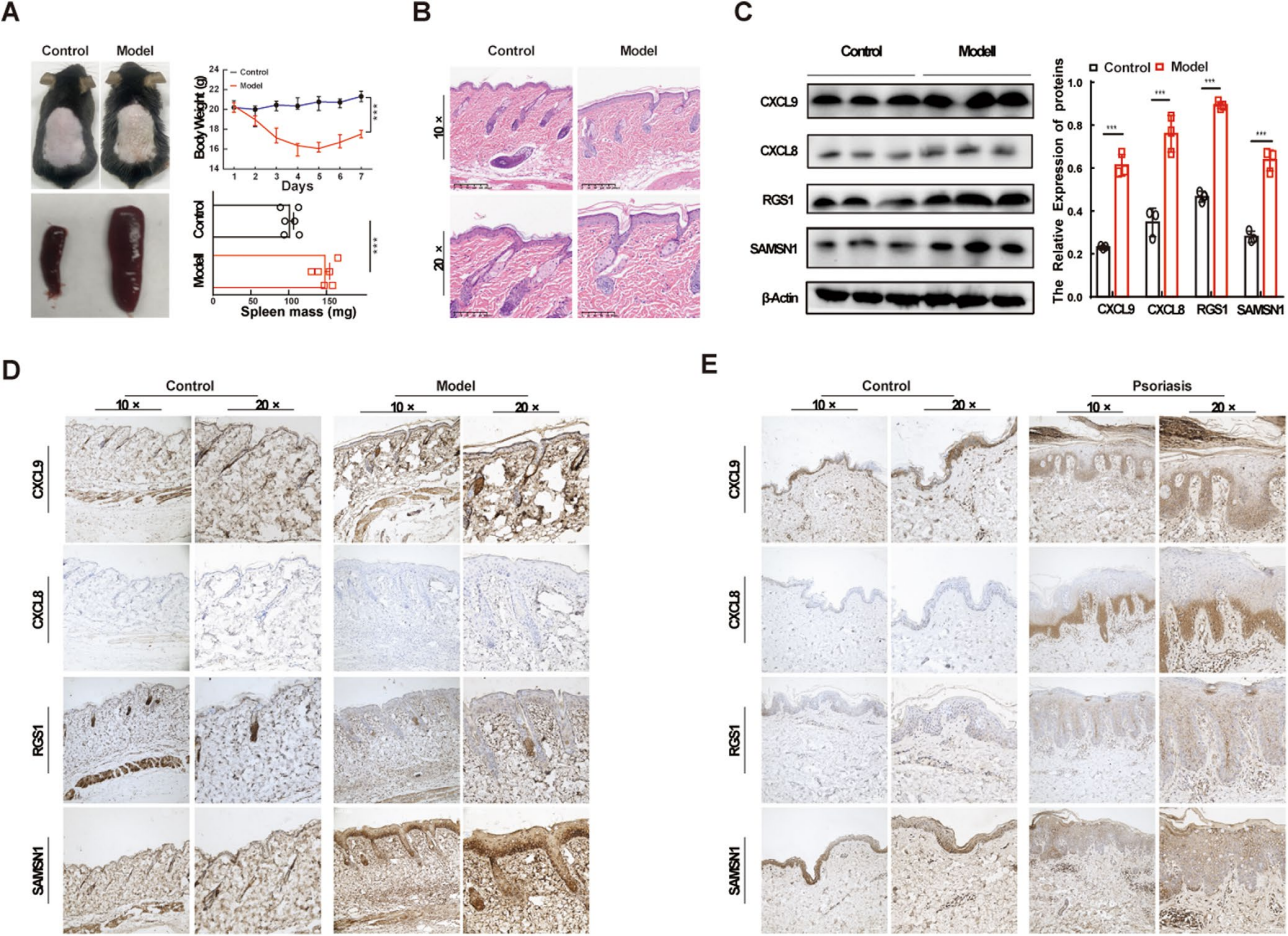
Expression validation of ImmDEGs. (A) Representative images of imiquimod‐induced psoriasis‐like skin inflammation in mice and their spleen. Statistics show the body weight and spleen weight in each group. (B) Representative images of H&E staining showing the skin tissue pathology of control and psoriasis mice. (C) The protein levels of CXCL9, CXCL8, RGS1, and SAMSN1 in each group. *n* = 3 in each group. ***p < 0.001 vs. control. (D,E) Immunohistochemistry staining showing the histopathology and expression of CXCL9, CXCL8, RGS1, and SAMSN1 in psoriatic mice and patient skin.

Imiquimod (IMQ) cream was smeared on the shaved back skin of mice for 7 consecutive days to induce psoriasis‐like skin inflammation in mice models. As expected, IMQ treatment mice exhibited typical psoriatic characteristics with significant pathological alterations, including significant parakeratosis, redness, scaling and thickening, acanthosis, and perivascular infiltration of inflammatory cells in the upper dermis, a phenotype typical of human psoriatic skin compared with healthy controls, while the mice body weight was obviously increased, and spleen weight was significantly reduced compared with control (Figure 6A). HE staining showed the skin pathological changes of different groups (Figure 6B). Western blot indicated that the expression of RGS1, SAMSN1, CXCL8, and CXCL9 in psoriasis‐like mice skin tissues was significantly upregulated (Figure 6C). Furthermore, immunohistochemistry data showed that the expression levels of RGS1, SAMSN1, CXCL8, and CXCL9 were all significantly increased in clinical psoriatic skin tissue and psoriasis‐like lesion skin tissue (Figure 6D,E). Taken together, our study validated the bioinformatics findings both in clinical tissue specimens and psoriasis‐like mice models.

## 4 Discussion

Psoriasis is a chronic inflammatory skin disease affecting approximately 2%–3% of the global population, imposing a substantial burden on individuals and society. Characterized by relapsing–remitting course and pathological features of high immune cell infiltration with histological alterations in lesional skin [45] it remains incompletely understood in terms of etiological factors and pathogenesis. Growing experimental and clinical evidence highlights the critical role of crosstalk between the innate and adaptive immune systems in driving or regulating psoriatic development. Thus, investigating the role of immune‐related genes in psoriasis progression is imperative. Identifying immune cell infiltration‐related biomarkers from lesional tissues may not only improve diagnosis, monitoring, and treatment but also advance our understanding of its pathogenesis.

In this study, we analyzed 11 psoriasis‐related mRNA microarray datasets (GEO database), comprising 605 psoriatic plaque samples and 611 normal samples. These datasets were integrated into a GEO cohort to systematically identify immune‐related genes associated with psoriasis. GO and KEGG enrichment analyses confirmed the close association of both innate and adaptive immune systems with the maintenance of psoriatic inflammation. Subsequently, we evaluated the infiltration levels of 28 immune cell types in the PIME and stratified lesional skin samples into two subtypes (C1 and C2). WGCNA revealed a significant association between the “black” module and these two subtypes. By intersecting DEGs between PP and PN samples, DEGs between C1 and C2 subtypes, and hub immune‐related genes in the “black” module, five genes—CXCL8, CXCL9, RGS1, CCL18, and SAMSN1—were identified as ImDEGs.

To validate these findings, we analyzed lesional skin tissues from psoriasis patients and imiquimod (IMQ)‐induced psoriatic mouse models. Expression levels of CXCL8, CXCL9, SAMSN1, and RGS1 were significantly upregulated in lesional tissues from both patients and mouse models. Previous studies have reported upregulated CCL18 in psoriatic lesional skin [46, 47]. Diagnostic efficacy of the five ImDEGs was evaluated via ROC curve analysis, which indicated favorable performance for all ImDEGs. In our study, the IMscore model was primarily derived and tested using retrospective GEO microarray datasets, with preliminary validation in a limited set of human/murine tissue samples. Although the model demonstrated promising performance in internal validation, it has not yet been independently validated in large‐scale external cohorts using different platforms. The IMscore demonstrates high diagnostic potential in our cohort (AUC = 0.979). However, several key challenges must be addressed to advance its translational application. First, while our histological and molecular data confirm the expression of key genes (CXCL8, CXCL9, SAMSN1, RGS1) within lesions, these methods lack single‐cell resolution for precise immune cell quantification. Future studies employing flow cytometry or single‐cell RNA sequencing are needed to validate the predicted immune infiltration dynamics at a cellular level. Second, the model requires validation in independent, publicly available datasets to confirm its generalizability. Finally, prior to clinical adoption, rigorous steps including technical standardization of the assay, robust prospective clinical validation, and thorough cost‐effectiveness analyses are imperative.

Chemokines, a class of secreted small‐molecule proteins, play a critical role in leukocyte chemotaxis and inflammatory response initiation. Classified into four subfamilies (C, CC, CXC, and CX3C) based on N‐terminal cysteine residues, they regulate recruitment and activation of leukocytes (T cells, macrophages, neutrophils) within the PIME [48]. CXCL8, A potent neutrophil chemoattractant, CXCL8 is strongly linked to neutrophil infiltration in psoriatic skin [49]. It recruits and activates neutrophils, thereby promoting inflammatory pathway activation in the PIME [50]. A study has show that TOPK activates STAT3/NF‐κB to upregulate neutrophil chemoattractants (CXCL1/2/8), leading to massive neutrophil infiltration [51]. CXCL9, another CXC subfamily member, CXCL9 is similarly upregulated in psoriatic skin [52]. CXCL9 is not just a generic inflammation marker; it is a direct transcriptional target of the pathogenic STAT3 pathway in keratinocytes and a functional mediator of immune cell recruitment [53]. Our study also confirmed CXCL9 association with immune cell infiltration. CCL18, A CC subfamily chemokine, CCL18 interacts with CCR8 to mediate T‐cell homing in inflammatory skin lesions [54]. Upregulated CCL18 is linked to allergic contact hypersensitivity and chronic inflammatory disorders (e.g., atopic dermatitis) [55]. Kim et al. reported significant upregulation of CCL18 mRNA in psoriatic plaques [56], and Purzycka‐Bohdan et al. observed elevated serum CCL18 levels in psoriasis patients [57]. In our study, CCL18 expression positively correlated with infiltration levels of multiple immune cells, particularly T‐cell subtypes. These findings underscore the systemic roles of chemokines in the psoriatic immune microenvironment. Therefore, investigating the intricate mechanisms of chemokine‐PIME interaction represents a promising area for future research.

The regulators of G protein signaling (RGS) are a family of cellular proteins that deactivate G proteins by accelerating their intrinsic GTPase activity, thereby inhibiting downstream signaling of G protein‐coupled receptors (GPCRs) for chemokines [58]. Notably, RGS1, a key RGS family member, plays a critical role in inflammatory responses. It is dysregulated in multiple immune diseases [59] and influences B cells, T cell, NK cell, and macrophage infiltration [60, 61]. Additionally, RGS1 is highly expressed in psoriatic skin lesions and has been proposed as a potential diagnostic biomarker for psoriasis [62]. These results align with our bioinformatic findings (Figure 5F). RGS1 has not been a primary focus of study in the context of psoriasis. However, its functional roles have been implicated in other inflammatory diseases. For instance, in rheumatoid arthritis, RGS1 is involved in modulating Toll‐like receptor signaling pathways [63]. In the context of airway immunity, it contributes to maintaining immune balance through the regulation of calcium signaling [64]. Building upon these established roles in distinct inflammatory settings, a novel, testable hypothesis emerges for psoriasis: RGS1 may contribute to psoriatic pathogenesis by dysregulating GPCR‐mediated signaling networks within resident or infiltrating immune cells. This dysregulation could, in turn, perturb critical cellular processes such as activation thresholds and trafficking, thereby influencing the local immune microenvironment.

SAMSN1, also known as HACS1, SLy2, or NASH1, is a member of a novel gene family of putative adaptor and scaffold proteins [65]. It is preferentially expressed and primarily regulates inflammatory responses in various immune cell [66]. Zhu et al. [67] reported that SAMSN1 is upregulated in activated B cells and promotes their differentiation into plasma cells. In addition, Amend et al. showed that loss of SAMSN1 in macrophages enhanced M2 polarization [68]. To our knowledge, SAMSN1 has not been previously reported in the context of psoriasis. However, a recent study revealed that in a sepsis model, SAMSN1 induces immunosuppression by promoting the expression of co‐inhibitory molecules on macrophages via the KEAP1‐NRF2 signaling pathway, subsequently leading to T‐cell exhaustion [69]. Based on this emerging role in immunoregulation, we hypothesize that in psoriasis, SAMSN1 may similarly function as a novel modulator of lymphocyte interactions, potentially influencing the cross‐talk between T cells and macrophages within the psoriatic inflammatory microenvironment. In our study, SAMSN1 was identified as a psoriasis risk factor, with its expression correlating with infiltration levels of T cells, B cells, macrophages, and neutrophils. However, the relationship between SAMSN1 expression and the PIME requires further investigation.

The five ImDEGs were used to construct a diagnostic prediction model named IMScore. ROC curves and forest plots confirmed the robust predictive efficacy of IMScore (Figure 5G). Additionally, IMScore exhibited strong correlations with multiple immune cell subtypes, particularly T cells, in the PIME. GSEA further validated the key roles of these five ImDEGs in psoriasis pathogenesis.

In conclusion, this study identified five immune‐related genes potentially involved in promoting psoriatic inflammation. These findings may contribute to improving therapeutic approaches for psoriasis in clinical practice.

## Author Contributions

Tingjin Zheng, Rong Xu, and Jianming Zhang performed and analyzed the experiments. Hao Huang collected the raw data. Hongfeng Tang and Chong Zeng conceived and designed the study. Zhishan Zhang wrote the paper. Chong Zeng and Hao Huang helped us improving the style of writing English. All authors read and approved the final manuscript. All authors have agreed to be accountable for all aspects of this work.

## Ethics Statement

This study was carried out by analyzing data from the medical record management system of the Shunde Hospital, Southern Medical University (The First People's Hospital of Shunde) in Foshan, Guangdong Province China. The study was approved by the ethics committee of the hospital.

## Consent

All authors consent this manuscript for publication.

## Conflicts of Interest

The authors declare no conflicts of interest.

## Supporting information


**Figure S1:** Batch effects removal for GEO datasets. (A) Distribution boxplot of datasets before batch processing. (B, C) PCA diagram of datasets before batch processing. (D) Distribution boxplot of CDs after batch processing. (E, F) PCA diagram of CDs after batch processing.

**Table S1:** Baseline information regarding the selected datasets.

**Table S2:** The identified DEGs between PN and PP samples.

**Table S3:** The comprehensive details pertaining to GO are exhibited.

**Table S4:** The comprehensive details pertaining to the GSEA are presented.

**Table S5:** The 28 immune cells infiltration in each psoriasis samples.

**Table S6:** The identified DEGs between C1 and C2 subcluster of PP.

**Table S7:** The WGCNA identified Immune‐related hub genes.

**Table S8:** The details pertaining to the WGCNA identified immune‐related genes.

## Data Availability

The data that support the findings of this study are available from the corresponding author upon reasonable request.
