# Supplementary figures and images for: Experimental Validation of an Immune Cell Infiltration Signature in Psoriasis: Translating Computational Modeling to In Vivo Efficacy
Source: J Clin Lab Anal. 2026 Mar 19;40(8):e70203. doi: 10.1002/jcla.70203 (PMC13107425; doi:10.1002/jcla.70203)

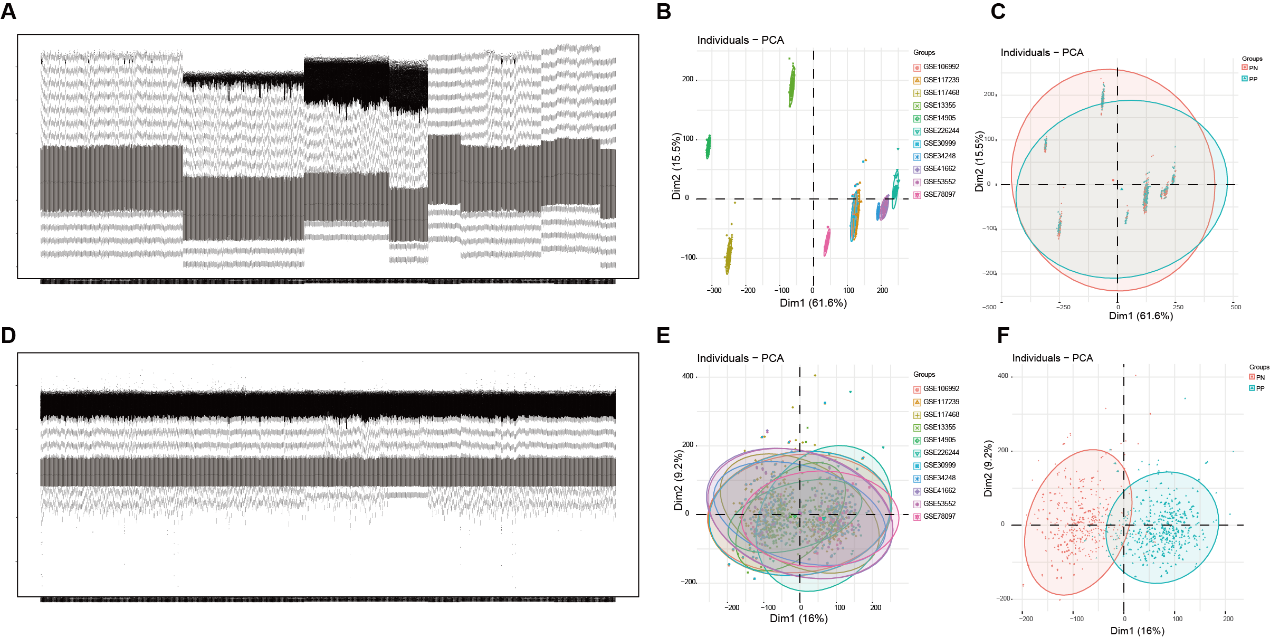

Supplement: Supplementary file 1 — Figure S1: Batch effects removal for GEO datasets. (A) Distribution boxplot of datasets before batch processing. (B, C) PCA diagram of datasets before batch processing. (D) Distribution boxplot of CDs after batch processing. (E, F) PCA diagram of CDs after batch processing. [file JCLA-40-e70203-s002.zip › jcla70203-sup-0001-FigureS1@Supplementary Figure 1.png]
